# **Editorial:** Resistant starch: advances and applications in nutrition for disease prevention

**DOI:** 10.3389/fnut.2025.1636551

**Published:** 2025-06-19

**Authors:** Hongmin Dong, Xianyang Bao, Hongliang Zeng

**Affiliations:** ^1^Department of Food Science, Cornell University, Ithaca, NY, United States; ^2^John A. Paulson School of Engineering and Applied Sciences, Harvard University, Cambridge, MA, United States; ^3^Department of Food Science, Fujian Agriculture and Forestry University, Fuzhou, China

**Keywords:** resistant starch (RS), gut micobiome, glycemic control, food processing, metabolic health, chronic disease prevention, starch modification, short-chain fatty acid (SCFA)

With the escalating global burden of chronic diseases including type 2 diabetes, cardiovascular disease, obesity, and inflammatory conditions, there is increasing interest in dietary strategies that extend beyond simple caloric reduction. Among emerging bioactive components, resistant starch (RS) has gained considerable attention due to its unique physiological functions and broad applications in nutrition and health. This Research Topic, “*Resistant Starch: Advances and Applications in Nutrition for Disease Prevention*,” brings together 10 peer-reviewed articles that collectively present a multidisciplinary and up-to-date exploration of RS. These contributions offer critical insights into its molecular characteristics, metabolic effects, food applications, and future directions in research and functional food innovation.

## Molecular classification and structural characteristics

The foundation of understanding RS is rooted in its classification and molecular structure. RS is typically classified into five primary types: RS1 (physically inaccessible starch), RS2 (native granular starches such as high amylose maize), RS3 (retrograded starch formed by cooking and cooling), RS4 (chemically modified starch), and RS5 (amylose lipid complexes). Among these, RS5 has gained increasing interest due to its stability, resistance to digestion, and potential physiological benefits. Studies in this Research Topic highlight how preserving starch crystallinity and molecular architecture is crucial for digestion resistance and fermentation patterns in the colon (Baptista et al.). These structural insights guide the design of targeted modifications to starch, optimizing RS formation and enhancing its functional potential in food products (Zhang et al.; Warwate et al.). One study investigated the synergistic modification of rice starch using hot-melt extrusion and nobiletin, a citrus-derived polymethoxylated flavone (Zhang et al.). This combined approach resulted in enhanced molecular interactions and altered multi-scale structures, resulting in increased thermal stability and reduced *in vitro* digestibility of starch. The application of such dual-modification techniques offers promising directions for tailoring the functional properties of starch-based ingredients, including increasing RS content and reducing glycemic impact in functional food formulations.

## Physiological effects and gut microbiome interactions

Several studies in this Research Topic confirmed that intake of RS, particularly RS1 and RS2, improves glycemic control by lowering postprandial glucose and fasting insulin levels (Kaur et al.; Chauhan et al.). This effect is highly relevant for the management and prevention of type 2 diabetes and related metabolic conditions. Furthermore, RS acts as a prebiotic, selectively stimulating the growth of beneficial gut bacteria such as *Bifidobacterium, Faecalibacterium prausnitzii*, and *Akkermansia muciniphila*. This microbial modulation enhances SCFA production, particularly butyrate, which has been linked to anti-inflammatory effects, improved gut barrier function, and even modulation of systemic immune responses. One clinical trial using a resistant starch blend from potato, banana, and apple fibers demonstrated improved gastrointestinal symptoms and favorable shifts in microbiome composition, demonstrating RS's potential in human health interventions (Hanes et al.). These findings highlight the role of individual microbiome variability in influencing RS fermentation and health outcomes, emphasizing the need for personalized nutrition approaches.

## Food processing and technological applications

The translation from laboratory research to commercial application depends heavily on understanding how food processing affects RS content. Processing methods such as milling, heating, fermentation, and cooling induce changes in starch structure and consequently RS levels. Several studies in this Research Topic investigate how traditional and novel cooking and storage methods impact RS content in staple foods. For example, research on commonly consumed Indian wheat products demonstrates that cooking techniques like boiling and shallow frying increase RS levels, while deep frying reduces them. Storage conditions, especially refrigeration, promote starch retrogradation, thereby increasing RS content (Kaur et al.). Similarly, mung beans subjected to specific cooking and storage regimes showed increased RS content and favorable metabolic outcomes *in vivo* (Chauhan et al.). Such findings offer practical guidance for food manufacturers and consumers aiming to maximize RS intake through everyday foods. Food technologists also explore the creation of RS-enriched staples such as bread, pasta, and rice by incorporating high-amylose or modified starches. Challenges related to dough manipulation, sensory characteristics, and consumer acceptance are addressed, demonstrating that RS enrichment can lower the glycemic index of foods without compromising taste or texture (Warwate et al.). These innovations offer practical strategies for delivering RS's health benefits through everyday diets.

## Broader metabolic and health implications

Beyond glucose regulation and gut health, RS impacts diverse metabolic and immunological pathways. It modulates bile acid metabolism, gut immune responses, and systemic inflammatory markers such as C-reactive protein and interleukins. Randomized controlled trials report reductions in LDL cholesterol and systemic inflammation with RS supplementation, suggesting benefits that extend to cardiovascular risk reduction and weight management (Wan et al.). The link between RS intake and weight regulation is particularly notable. Clinical data indicate that RS enhances satiety and appetite control, contributing to modest reductions in body weight and fat mass, especially in overweight or diabetic populations. These findings position RS as an adjunctive nutritional strategy in combating obesity and related metabolic disorders. Importantly, epidemiological evidence from large cohort studies associates higher RS intake with reduced all-cause and cancer-specific mortality, highlighting its potential role in long-term health and longevity (Wan et al.). These associations warrant further mechanistic and interventional studies to confirm causality and elucidate optimal intake levels.

## Emerging frontiers and future directions

This Research Topic concludes with promising perspectives on novel RS complexes, such as starch-protein and starch-polyphenol conjugates. These innovative structures exhibit improved stability and enhanced physiological effects, broadening the functional repertoire of RS. One study highlights the synergistic effect of dietary amylose-to-amylopectin ratio on antioxidant status and amino acid metabolism in piglets, showing how starch structure interacts with nutrient metabolism in the liver (Yang et al.). Additionally, new research explores the role of mineral intake in brain health, including associations between manganese, zinc, magnesium, and cognitive performance, which may intersect with RS's influence on gut-brain axis and metabolic regulation (Chen et al.). Furthermore, a study from the Iranian Teachers Cohort reported that higher dietary glycemic index and load were significantly associated with increased odds of osteoporosis, independent of insulin-related dietary measures. These findings emphasize the importance of carbohydrate quality in bone health and suggest that RS, by lowering glycemic response and supporting SCFA production, could potentially contribute to the mitigation of osteoporosis risk (HoushiarRad et al.).

Future studies should continue to elucidate mechanistic pathways underlying RS's health effects and explore personalized nutrition strategies that consider individual microbiome and metabolic profiles. Moreover, optimizing food processing technologies to maximize RS content while ensuring consumer acceptance will be crucial for translating research findings into impactful public health solutions.

## Conclusion

This Research Topic provides a timely and comprehensive examination of resistant starch, highlighting its complex molecular characteristics, physiological benefits, interactions with gut microbiota, and practical food applications. As a versatile dietary component with the potential to improve metabolic health, reduce inflammation, and support chronic disease prevention, RS represents an important focus in contemporary nutrition science. The integration of fundamental research with food technology innovation offers a promising path toward leveraging resistant starch for enhanced public health worldwide.

